# Initiator and executioner caspases in salivary gland apoptosis of *Rhipicephalus haemaphysaloides*

**DOI:** 10.1186/s13071-020-04164-5

**Published:** 2020-06-05

**Authors:** Yanan Wang, Shanming Hu, Mayinuer Tuerdi, Xinmao Yu, Houshuang Zhang, Yongzhi Zhou, Jie Cao, Itabajara da Silva Vaz, Jinlin Zhou

**Affiliations:** 1 Key Laboratory of Animal Parasitology of Ministry of Agriculture, Shanghai Veterinary Research Institute, Chinese Academy of Agricultural Sciences, Shanghai, 200241 China; 2 Centro de Biotecnologia, Universidade Federal do Rio Grande do Sul, Porto Alegre, RS Brazil

**Keywords:** Salivary gland degeneration, Transcriptome, Apoptosis, Caspase

## Abstract

**Background:**

Apoptosis is fundamental in maintaining cell balance in multicellular organisms, and caspases play a crucial role in apoptosis pathways. It is reported that apoptosis plays an important role in tick salivary gland degeneration. Several different caspases have been found in ticks, but the interactions between them are currently unknown. Here, we report three new caspases, isolated from the salivary glands of the tick *Rhipicephalus haemaphysaloides*.

**Methods:**

The full-length cDNA of the RhCaspases 7, 8 and 9 genes were obtained by transcriptome, and RhCaspases 7, 8 and 9 were expressed in *E. coli*; after protein purification and immunization in mice, specific polyclonal antibodies (PcAb) were created in response to the recombinant protein. Reverse-transcription quantitative PCR (RT-qPCR) and western blot were used to detect the existence of RhCaspases 7, 8 and 9 in ticks. TUNEL assays were used to determine the apoptosis level in salivary glands at different feeding times after gene silencing. The interaction between RhCaspases 7, 8 and 9 were identified by co-transfection assays.

**Results:**

The transcription of apoptosis-related genes in *R. haemaphysaloides* salivary glands increased significantly after tick engorgement. Three caspase-like molecules containing conserved caspase domains were identified and named RhCaspases 7, 8 and 9. RhCaspase8 and RhCaspase9 contain a long pro-domain at their N-terminals. An RT-qPCR assay demonstrated that the transcription of these three caspase genes increased significantly during the engorged periods of the tick developmental stages (engorged larval, nymph, and adult female ticks). Transcriptional levels of RhCaspases 7, 8 and 9 in salivary glands increased more significantly than other tissues post-engorgement. RhCaspase9-RNAi treatment significantly inhibited tick feeding. In contrast, knockdown of RhCaspase7 and RhCaspase8 had no influence on tick feeding. Compared to the control group, apoptosis levels were significantly reduced after interfering with RhCaspase 7, 8 and 9 expressions. Co-transfection assays showed RhCaspase7 was cleaved by RhCaspases 8 and 9, demonstrating that RhCaspases 8 and 9 are initiator caspases and RhCaspase7 is an executioner caspase.

**Conclusions:**

To the best of our knowledge, this is the first study to identify initiator and executioner caspases in ticks, confirm the interaction among them, and associate caspase activation with tick salivary gland degeneration.
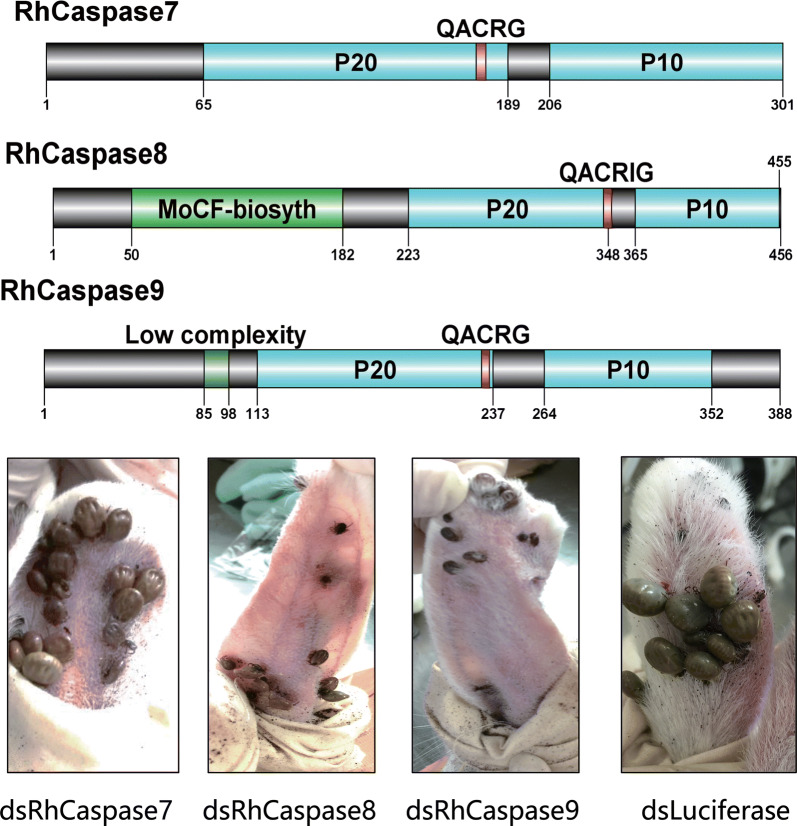

## Background

Tick species feed on a wide range of hosts and are important vectors of infectious agents [1–3]. Ticks also may remain attached to the host, feeding on host blood, for long periods [4]. Tick-borne pathogens (TBPs) are transmitted to hosts through tick bites assisted by saliva [5, 6].

Programmed cell death (PCD) is a conserved phenomenon, present in eukaryotes and several prokaryotes, and it plays a fundamental role in cell homeostasis [7–9]. PCD is involved in a series of processes and modifications, such as sculpting organs and tissues and removing unwanted structures and damaged cells [10]. Apoptosis is an important component of PCD [11, 12]. Several studies have attempted to investigate these mechanisms. For example, L’Amoreaux et al. [13] used terminal deoxynucleotidyl transferase dUTP nick-end labeling (TUNEL) staining to show that the rate of DNA fragmentation was significantly higher in degenerated salivary glands. In addition, Freitas et al. [14] showed that caspase 3 activity levels increased significantly in tick salivary glands 72 h post-engorgement.

Caspases are members of the cysteine-1 aspartate-specific protease family [15, 16]. There are major groups of caspases in mammals, including initiator caspases and executioner caspases. Caspases 2, 8, 9, 10, 11 and 12 are known to be initiators, and caspases 3, 6 and 7 are known to be executioners [17, 18]. Usually, caspases are present in cells as zymogens and require proteolytic cleavage to be converted into active enzymes [18, 19]. The caspase-associated recruitment domain (CARD) is an interaction motif between different proteins, which plays critical roles in regulating the activation of caspase and nuclear factor-κB (NF-κB) in the context of apoptosis and inflammation [20]. At the beginning of apoptosis, initiator caspases (such as caspase 2 and caspase 9) are activated through the CARD domain and then generate a cascade reaction which activates downstream executioner caspases leading to completion of the apoptosis processes [21]. All caspases are formed by two subunits, a large subunit (p20) and a small subunit (p10). They also have a conserved active-site motif (QACXG; where X is R, Q or G) in the large subunit which mediates the cleavage of proteins at peptide bonds involving the carbonyl group of aspartate residues.

In addition to the well-established role of caspases in apoptosis, there is increasing evidence that caspases have additional functions in several biological events [22]. For example, caspase 1 and caspase 11 play a role in inflammation and in mediating inflammatory cell death caused by pyroptosis [23, 24]. Similarly, caspase 8 plays a dual role in cell death, mediating both receptor-mediated apoptosis and necroptosis. Caspase 3 is involved in tissue differentiation, regeneration, and neural development in a unique manner that does not involve any apoptotic activity [25–28]. Caspase 2, 8 and 14 have demonstrated anti-tumor roles [29, 30]. Caspase 2 is unique with putative roles in maintaining genomic stability, metabolic control, autophagy and aging [31, 32].

Caspases are highly conserved in most organisms, from bacteria to mammals. Although relatively well known in many organisms, information about caspases in ticks is scarce [33–35]. Since the roles of caspases in apoptosis are well known in animals, we postulated they might have a similar role in ticks [33–36]. However, in ticks and tick-borne diseases, the roles of caspases are poorly understood. We used transcriptional analysis to compare tick salivary glands at different feeding times. Transcription of apoptosis-related proteins increased after tick engorgement. Caspase functions were investigated using an RNAi approach in the salivary glands of engorged *Rhipicephalus haemaphysaloides*.

## Methods

### Ticks and tissue collection

Adult *R. haemaphysaloides* were collected from Wuhan Hubei Province and kept and fed in our laboratory. Different tick developmental stages were attached to the ears of 9–12-week-old female New Zealand White rabbits (SLAC, Shanghai Institutes for Biological Science, CAS, Shanghai, China) using ear bags. After microdissection, tick tissues were washed twice with cold phosphate-buffered saline (PBS, pH 7.4, with 0.14 M NaCl and 0.0027 M KCl, 0.01 M phosphate buffer; Gibco, Life Technologies, Carlsbad, CA, USA) and stored at − 80 °C in PBS or TRIzol reagent (Invitrogen, Carlsbad, CA, USA).

### RNA extraction and cDNA synthesis

RNA from ticks at different development stages and different tissues from the adult female ticks during different feeding times was isolated using TRIzol reagent (Invitrogen). The synthesis of first-strand cDNA was performed using a HiScript® III RT SuperMix for qPCR (+gDNA wiper) kit (Vazyme Biotech, Nanjing, China) according to the manufacturer’s protocol.

### Transcriptome of *R. haemaphysaloides* salivary glands

Salivary glands of both unfed and fed (engorged) *R. haemaphysaloides* females were homogenized in TRIzol reagent (Invitrogen), and purified RNA was used for the construction of paired-end cDNA libraries using a NEBNext® Ultra^TM^ RNA Library Prep Kit (New England Biolabs, Ipswich, MA, USA), according to the manufacturerʼs instructions. Sequences were tagged with specific barcodes and paired-end reads were sequenced using an Illumina HiSeq platform (Illumina, San Diego, CA, USA) at the Beijing Genomics Institute (BGI, Beijing, China).

RNAseq data were cleaned and formatted using an Agilent 2100 Bioanalyzer (Agilent Technologies, Santa Clara, CA, USA). The high-quality reads were assembled by the Trinity program using default parameters [37]. The assembled transcripts were extended and clustered using TGICL software [38]. The assembled transcripts were processed for further functional annotation and classification analysis. The *de novo* approach for transcriptome assembly, TransDecoder (http://transdecoder.sourceforge.net), was used to identify putative CDS sequences from the contigs. Seven different function databases (NR, NT, Gene Ontology (GO) terms [39], Clusters of euKaryotic Orthologous Groups (KOG) [40], Kyoto Encyclopedia of Genes and Genomes (KEGG) pathways [41], SwissPprot and InterPro [42]) were used to annotate all of the assembled transcripts (Unigene). Differentially expressed genes were identified using the MA-plot-based method with a random sampling model by comparing the unfed library to the engorged library. Genes with fold change *>* 3 and a *P*-value *<* 0.001 were considered as differentially expressed genes.

### Cloning, sequence analysis and expression of Rhcaspases 7, 8 and 9

Three caspase-like molecules with conserved caspase domains were identified and named RhCaspase 7, 8 and 9. Cloning primers of RhCaspases 7, 8 and 9 (Additional file 1: Table S1) were designed according to ORF regions found in contig sequences assembled in the salivary gland transcriptomes of unfed and engorged *R. haemaphysaloides*. The amplicons with complete ORF sequences were ligated to pMD-18T. SignalP 4.1 (http://www.cbs.dtu.dk/services/SignalP/) [43] and ExPASy (http://web.expasy.org/compute pi/) [44] were used for the signal peptide analysis and isoelectric point (PI) prediction. Similar sequences of target genes were searched using the BLASTp server (National Center for Biotechnology Information, National Institute of Health). The sequences of RhCaspases 7, 8 and 9 were aligned with caspases of other species by Genetyx ver. 6 (Genetyx, Tokyo, Japan). For phylogenetic analysis, the alignment of the sequences was performed using the MUSCLE algorithm [45] and inferred using the maximum likelihood method with the default settings in MEGA X software [46]. Bootstrap support was estimated using 500 bootstrap replicates. The arthropod caspase sequences were obtained from the manually curated database CaspBase [47] and additional tick caspases were obtained from GenBank.

The accession numbers for sequences used are as follows: XM_029966994.1 (*Ixodes scapularis* caspase 7); XM_029970321.1 (*I. scapularis* caspase 3); KY056149.1 (*Locusta migratoria* caspase 9/Dronc). DQ666174.1 (*Haemaphysalis longicornis* caspase 2); DQ660369.1 (*H. longicornis* caspase 8); XM_029990833.1 (*I. scapularis* caspase 3 like); KT194090.1 (*R. haemaphysaloides* caspase 1); NP_001260718.1 (*Drosophila melanogaster* Ser/Thr-rich caspase, Dmel\Strica); NP_001303345.1 (*D. melanogaster* death associated molecule related to Mch2 caspase, Dmel\Damm); NP_476974.1 (*D. melanogaster* death caspase-1, Dcp-1); NP_477249.3 (*D. melanogaster* death related ced-3/Nedd2 like caspase; Dredd); NP_477462.1 (*D. melanogaster* death executioner caspase related to Apopain/Yama, Dmel\Decay); NP_524017.1 (*D. melanogaster* death regulator Nedd2 like caspase, Dmel\Dronc); NP_524551.2 (*D. melanogaster* death related ICE-like caspase, Dmel\Drice); XP_006570976.2 (*Apis mellifera* caspase 8); XP_016771440.1 (*A. mellifera* caspase Dronc); XP_394855.4 (*A. mellifera* caspase 3); XP_395697.2 (*A. mellifera* caspase 1); NP_001037050.1 (*Bombyx mori* caspase 1); NP_001182396.1 (*B. mori* caspase Nc); NP_001243935.1 (*B. mori* caspase 4); XP_001648537.2 (*Aedes aegypti* caspase-1); XP_001655433.2 (*Ae. aegypti* caspase Dronc); XP_001656809.1 (*Ae. aegypti* caspase); XP_021694895.1 (*Ae. aegypti* caspase 7); XP_021704883.1 (*Ae. aegypti* caspase); XP_021709829.1 (*Ae. aegypti* caspase 8); XP_021709830.1 (*Ae. aegypti* caspase 3 isoform); XP_021709833.1 (*Ae. aegypti* caspase 3); XP_021711617.1 (*Ae. aegypti* caspase 8); MN_395579 (*R. haemaphysaloides* caspase 7); ALQ_43547.1 (*R. haemaphysaloides* caspase 8); and MK_841509 (*R. haemaphysaloides* caspase 9).

### RT-qPCR analyses

The expression patterns of RhCaspases 7, 8 and 9 were analyzed in ticks feeding during different life stages (larva, engorged-larva, nymph, engorged-nymph, unfed male adult, fed male adult, unfed female adult and engorged female adult). The cDNAs of each stage were used as templates for RT-qPCR, with specific RhCaspase 7, 8 and 9 primers designed using Primer Premier 5 (Additional file 1: Table S2). RT-qPCRs were performed using ChamQ Universal SYBR qPCR Master Mix (Vazyme) green and gene-specific primers with a QuantStudio PCR System (Applied Biosystems, Austin, TX, USA). RT-qPCR cycling parameters were 95 °C for 30 s, followed by 40 cycles of 95 °C for 5 s and 60 °C for 30 s. All samples were analyzed three times.

The data were normalized to the gene expression of elongation factor-1 (*ELF1A*, GenBank: AB836665) [48] using the 2^−∆Cq^ method [49, 50]; ∆Cq was calculated by subtracting the average ELF1A Cq value from the average Cq value of the target gene.

### Expression of recombinant RhCaspases 7, 8 and 9 and antibody production

Recombinant RhCaspases 7, 8 and 9 expressed in an *Escherichia coli* system were used to obtain the sera. Specific RhCaspase 7, 8 and 9 primers were designed for cloning in pET-28a (Additional file 1: Table S3). The RhCaspase 7, 8 and 9 amplified-PCR products were purified and digested with *Bam*HI and *Hind*III (New England Biolabs) and ligated into pET-28a (Invitrogen) using In-Fusion HD Cloning Kits (Takara Clontech, Mountain View, CA, USA). Then, BL21 (DE3) (Tiangen, Beijing, China) were transformed with pET-28a-His-RhCaspases 7, 8 and 9. The positive clones were further screened by PCR using specific caspase primers. The expression of RhCaspases 7, 8 and 9 was performed by transforming the pET-28a-His-RhCaspase 7, 8 and 9 recombinant plasmids in *E. coli* BL21 (DE3). Isopropyl β-D thiogalacto pyranoside (IPTG) was added at a final concentration of 1 mM, and expression was induced at 20 °C for 12 h. After expression, the recombinant RhCaspases 7, 8 and 9 were affinity-purified under denaturation conditions using His agarose (Merck, Darmstadt, Germany).

For the first injection, 6–8-week-old BALB/c mice (SLAC, Shanghai Institutes for Biological Science, CAS) were immunized with 0.2 ml saline containing 100 μg His-RhCaspases 7, 8 and 9 mixed and emulsified with Freund’s complete adjuvant (Invitrogen) in an equal volume. For subsequent injections, Freund’s incomplete adjuvant (Invitrogen) instead of complete adjuvant was mixed with the same dose of antigen. The mixtures were inoculated 3 times, at 2-week intervals. Sera were collected three days after the third inoculation. The sera were stored at − 20 °C until use.

### Western blot

Total proteins of different development tick stages and different tick tissues were extracted using Tris-buffered saline (TBS; 10 mM Tris-HCl, pH 7.5; 150 mM NaCl with 1 mM phenylmethanesulfonyl fluoride). Total extracted protein amounts were determined using the Bradford Protein Assay Kit (Beyotime, Shanghai, China), following the manufacturer’s instructions. For SDS-PAGE (12%; Genescript, Nanjing, China), loading 20 µg protein/well was performed, and proteins on the gel were transferred onto a nitrocellulose membrane. The sera anti His-RhCaspase 7, 8 and 9 were used to detect caspases in protein extracts and an anti-tubulin primary antibody (Proteintech, Rosemont, IL, USA) was used as constitutive control to normalize the signal from the target protein. After primary incubations, the goat anti-mouse IgG (H + L) secondary antibody conjugated with HRP (Invitrogen), and IRDye 800CW goat anti-mouse IgG (H + L) (LI-COR, Nebraska, USA) were used as secondary antibodies in assays. Images were captured by ChemiDoc Touch (Bio-rad, Hercules, CA, USA) or Odyssey Imaging System (LI-COR).

### RNAi of Rhcaspases 7, 8 and 9

The RNAi experiments were designed against RhCaspase 7, 8 and 9 genes. For the design of RNAi primers, caspase sequences were screened by Primer Premier 5. Caspase-specific primers (Additional file 1: Table S4) containing the T7 polymerase promoter sequence were used for PCR amplification. The amplicons were then purified to obtain templates for double-stranded RNA synthesis using the T7 RiboMAX Express RNAi system (Promega, Madison, WI, USA). Unfed female ticks were microinjected with approximately 1 μg of dsRNA caspase. Control ticks were injected with unrelated dsLuciferase. The biological parameters analyzed were: attachment rate at 48 h; number of engorged ticks; and weight. RT-qPCR was used to evaluate gene silencing efficiency.

### TUNEL staining

Dissected salivary glands were fixed in 4% formalin and embedded in paraffin. Sections of salivary glands were mounted on microscope slides. Tissue sections were then deparaffinized, washed in 100% ethanol, and rehydrated. Samples were washed with PBS. After antigen retrieval with 0.1% Triton X-100, the tissues were incubated for 1 h with 1:9 TdT mixed with fluorescent-labeled dUTP at 37 °C, following the instructions of the Roche *in situ* Cell Death Detection Kit, POD (Roche, Mannheim, Germany). The cell nuclei were stained with 1 μg/ml 4’, 6’-diamidino-2-phenylindole (DAPI; Invitrogen) in distilled H_2_O for 20 min. After washing, the sections were mounted using Lab Vision^TM^ PermaFluor^TM^ (Invitrogen) medium under glass coverslips, then viewed and photographed on a Pannoramic DESK Digital Slide Scanner (3D Histech, Budapest, Hungary).

### Cells and transient co-transfection assays

HEK 293 cells were maintained in Dulbecco’s modified Eagle’s medium (DMEM; Gibco), supplemented with 8% heat-inactivated fetal bovine serum (Biological Industries, Kibbutz Beit Haemek, Israel) and 1% penicillin (Gibco) at 37 °C.

The full-length ORF of RhCaspases 7, 8 and 9 were inserted into the p3×Flag-CMV-14 vector (MiaoLing Plasmid Sharing Platform, Wuhan, China) with Flag tag at the N-terminal with gene-specific primers (Additional file 1: Table S3). Transfection using Lipofectamine™ 3000 Transfection Reagent (Invitrogen) was performed according to the manufacturer’s protocol with a DNA to Lipofectamine ratio of 1:2 w/v. The HEK 293 cells were transformed with 3 μg/well of plasmid or co-transfected with the equivalent amount of two different plasmids in 6-well plates.

### Data analysis

GraphPad PRISM 6.0 software (Graph Pad Software Inc., San Dieo, CA, USA) was used for all data analyses. Mean ± standard error (SE) values were calculated for three independent experiments, and two-tailed Student’s t-tests were used to identify significant differences between groups (*P < 0.05, **P < 0.01, ***P < 0.001, ****P < 0.0001).

## Results

### Transcriptome analysis of tick salivary glands

**Fig. 1:**
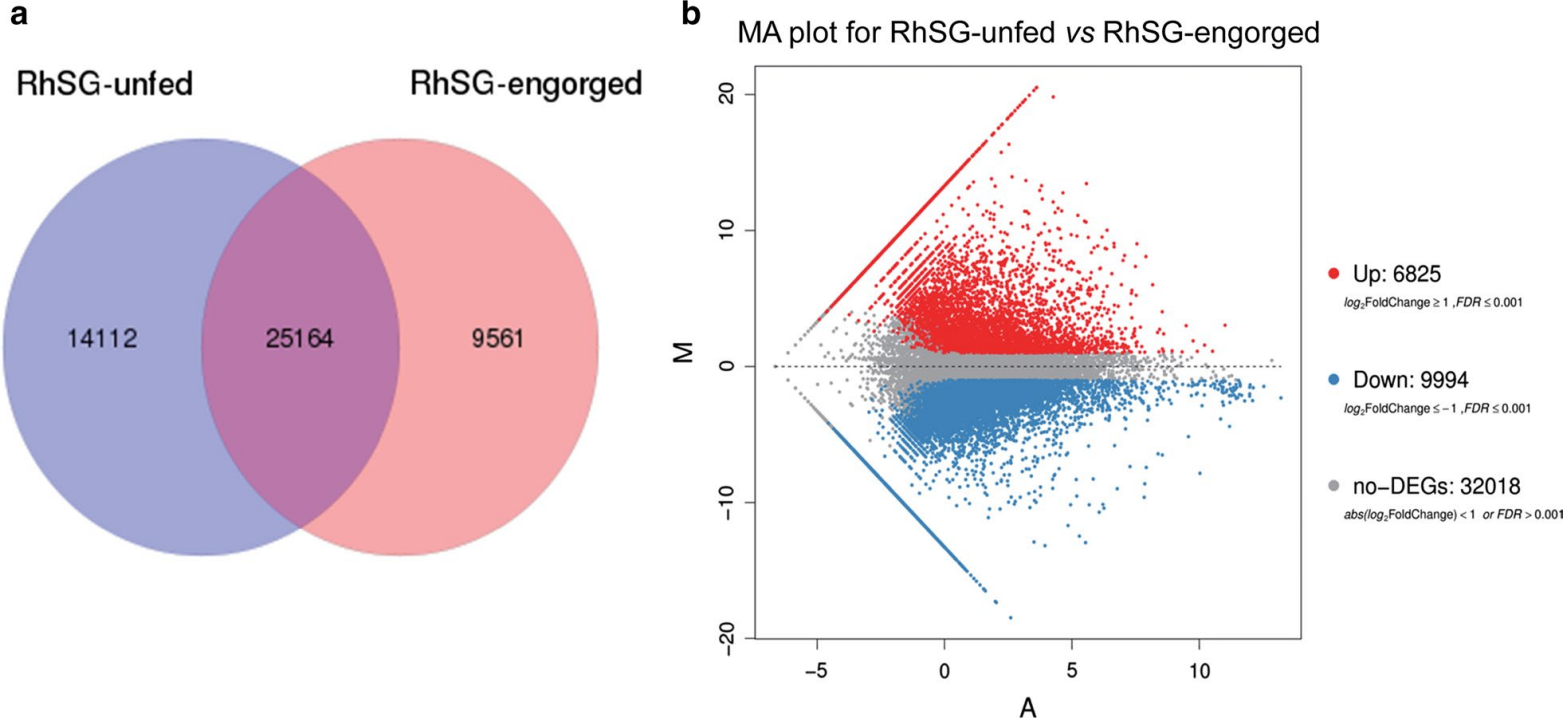
Transcriptome sequencing and differentially expressed gene analysis of unfed and engorged *Rhipicephalus haemaphysaloides* salivary glands. **a** Venn analysis of the transcriptome sequencing of unfed and engorged tick salivary glands. **b** Total number of differentially expressed genes (upregulated and downregulated) between unfed and engorged tick salivary glands

Unfed and engorged tick salivary glands were selected for transcriptome analysis and used to observe the expression of apoptosis-related genes. After RNAseq analyses, 65.38 and 44.16 Mb clean reads were obtained from unfed and engorged tick salivary glands, respectively. Reads were assembled and 39276 and 34725 unigenes were annotated from salivary glands of unfed and engorged ticks (Fig. 1a).

Using unfed tick salivary glands as a reference, 6825 and 9994 unique genes were upregulated and downregulated, respectively, in engorged tick salivary glands (Fig. 1b). The majority of the genes were expressed with log2 fold change values > 2 and ≤ 10 (about 95%). Genes showing values > 10 and ≤ 20 were less abundant (about 5%) (Fig. 1b). We deposited the RNAseq raw data to the NCBI SRA repository (accession numbers: SRR11523177 and SRR11523176). The enrichment GO terms (Additional file 2: Figure S1), Pheatmap-plot (Additional file 2: Figure S2), FASTA format sequence of unigenes (Additional file 3: Alignment S1) and differentially expressed genes lists (Additional file 4: Dataset S1) are provided as additional files.

### Both intrinsic and extrinsic apoptosis pathways were activated during tick salivary gland degeneration

**Fig. 2:**
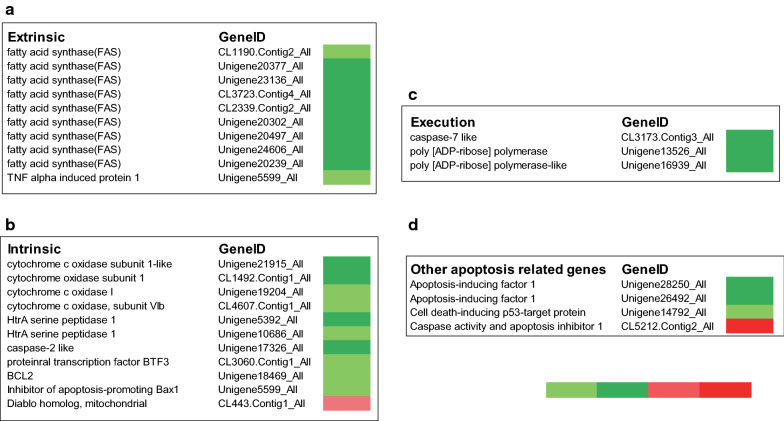
Expression of apoptosis related genes in *Rhipicephalus haemaphysaloides* salivary glands upregulated post-engorgement. **a** Intrinsic apoptosis pathway. **b** Extrinsic apoptosis pathway. **c** Executioner components. **d** Other apoptosis related genes. Light green, upregulated genes were selected as those between 2–10 fold (1 < log2 normalized fold change < 3.32); green, upregulated genes were selected as those expressed by more than 10 fold (log2 normalized fold change > 3.32); Pink, downregulated expressed gene were selected as those with a 2–10 fold (2 < log2 normalized fold change < 3.32); Red, downregulated expressed gene were those with a more than 10 fold (log2 normalized fold change > 3.32)

Caspase-dependent apoptosis pathways are divided into extrinsic and intrinsic pathways. Based on known sequences in other organisms, the RNAseq analysis of the tick salivary gland identified 28 apoptosis-related genes; 10 were related to the extrinsic apoptotic signaling pathway; and 11 were related to intrinsic apoptotic signaling pathway. Three were involved in the apoptosis execution phase and nine other genes were involved in apoptosis (Fig. 2a–d). Most of the apoptosis-related genes were upregulated after engorgement (Fig. 2a–d). These results suggest that apoptosis pathways have an important role in the degeneration of tick salivary glands. Based on the molecular analysis and expression profile, some representative components of apoptosis pathways were selected to characterize the tick salivary gland degeneration process.

### Identification of RhCaspase 7, 8 and 9

**Fig. 3:**
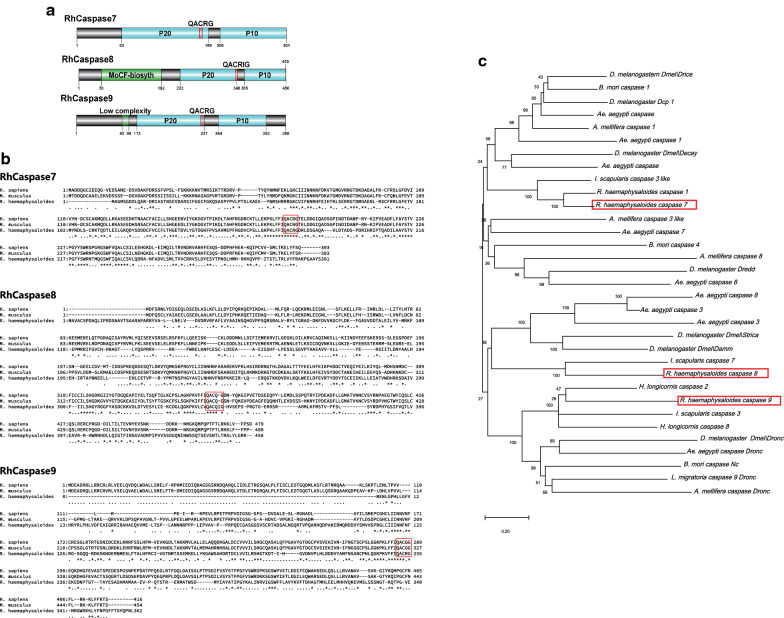
Sequence and structure analysis of *Rhipicephalus haemaphysaloides* RhCaspases 7, 8 and 9. **a** Domain structure of RhCaspases 7, 8 and 9. **b** Alignment of the deduced amino acid sequences of RhCaspases 7, 8 and 9. *Homo sapiens* caspase 7: P55210; *Mus musculus* caspase 7: P97864; *Homo sapiens* caspase 8: Q14790; *Mus musculus* caspase 8: O89110; *Homo sapiens* caspase 9: P55211; *Mus musculus* caspase 9: Q8C3Q9. **c** Phylogenetic tree of *R. haemaphysaloides* caspases with the caspases of other arthropod species. Bootstrap values after 500 simulations are shown at the branches and RhCaspases 7, 8 and 9 are marked with red rectangles

Using nucleic acid sequences obtained from RNAseq, specific cloning primers were designed according to the predicted sequences of *R. haemaphysaloides* caspase 7, 8 and 9. The ORF regions of the three caspase-related genes were cloned from the cDNA of fully engorged *R. haemaphysaloides* salivary glands and named RhCaspase 7, 8 and 9. RhCaspase7 ORF (GenBank: MN395579) has 903 bp encoding a protein of 301 amino acid residues (Fig. 3a, b) with a deduced molecular weight (MW) and theoretical isoelectric point (PI) of 34 kDa and 6.39, respectively. RhCaspase8 (GenBank: ALQ43547.1) and RhCaspase9 ORF (GenBank: MK841509) were also cloned, with 1368 bp and 1164 bp, respectively, and encoding proteins of 456 and 388 amino acid residues, respectively (Fig. 3a, b). They have deduced MWs of 51.7 kDa and 40.4 kDa, and PIs of 6.97 and 6.5, respectively. Sequence and structure analysis showed that they have similarity to mammal caspases. RhCaspase7, RhCaspase8, and RhCaspase9 had the conserved active site (QACR (I) G), a large subunit (p20 domain), and a small subunit (p10 domain), respectively. Similar to the executioner caspases, RhCaspase7 has a short prodomain in the N-terminus. In contrast, as in other initiator caspases, both RhCaspase8 and RhCaspase9 have a long N-terminal pro-domain (more than 90 amino acids). The pro-domain of RhCaspase8 named with MoCF-biosyth (involved in biosynthesis of molybdopterin cofactor). The structure of this domain is known, and it forms an α/β structure. In the known structure of gephyrin this domain mediates trimerisation. RhCaspase9 has a low complexity domain in the N-terminal, which is a molecular structural region of biased composition (Fig. 3a).

The phylogenetic analysis showed the relationship between tick caspases and caspases of other arthropods. Caspases 8 and 9 branched into one clade and caspase 7 branched to a different clade (Fig. 3c). All *R. haemaphysaloides* caspases grouped with similar arthropod caspases (Fig. 3c, Additional file 1: Figure S3).

### Expression of RhCaspases 7, 8 and 9 and production of antisera

The coding sequences of RhCaspases 7, 8 and 9 were cloned into prokaryotic expression vectors (pET-28a) to produce recombinant RhCaspases 7, 8 and 9. All of the recombinant proteins were expressed as inclusion bodies in *E. coli*. The recombinant proteins undergo a spontaneous cleavage as previously observed in recombinant caspases of *Spodoptera frugiperda* and *B. mori* [51, 52].

**Fig. 4:**
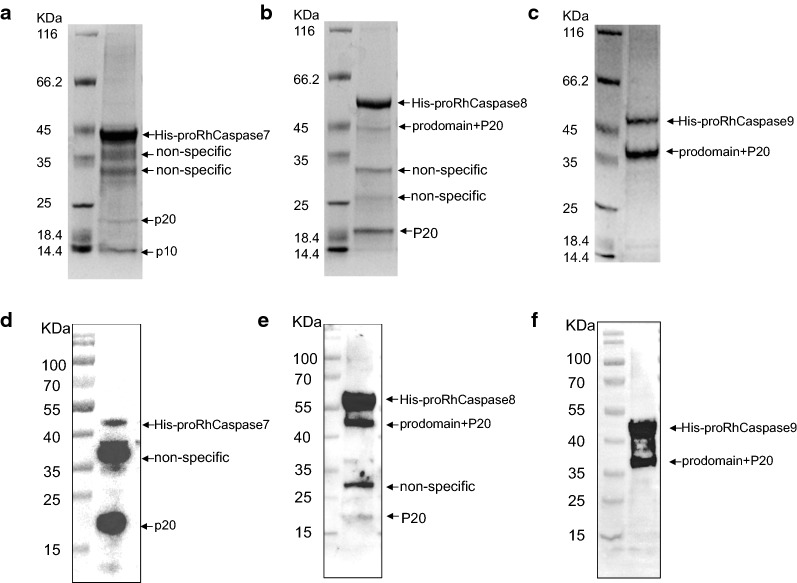
Expression of recombinant RhCaspases 7, 8 and 9. **a–c** Purification of recombinant RhCaspases 7, 8 and 9. Lane 1: M, the protein marker; Lane 2: recombinant protein after purification. **d–f** Western blot detection of RhCaspases 7, 8 and 9. Purified RhCaspases 7, 8 and 9 were immunoblotted with the anti-sera against RhCaspases 7, 8 and 9, respectively

After solubilisation and purification, His-RhCaspases 7, 8 and 9 (molecular weight: ~ 41 kDa, ~ 58.5 kDa and ~ 47.3 kDa were obtained, respectively (Fig. 4a–c). Initiator caspases had two cleavage sites, one located between the pro-domain and p20 and another between p20 and p10 (Fig. 4b, c). The MWs of proRhCaspases and cleaved-RhCaspases were similar to the corresponding mammalian caspase molecules. Purified recombinant RhCaspases 7, 8 and 9 were used to elicit polyclonal antibodies in rabbits. Western blot analysis (Fig. 4d–f) revealed that the three sera were able to identify the full recombinant proteins and the cleavage forms, and the fragment sizes were consistent with those observed by SDS-PAGE (Fig. 4a–f).

### Transcription and translation of caspase profiles in different stages, tissues and feeding periods

**Fig. 5:**
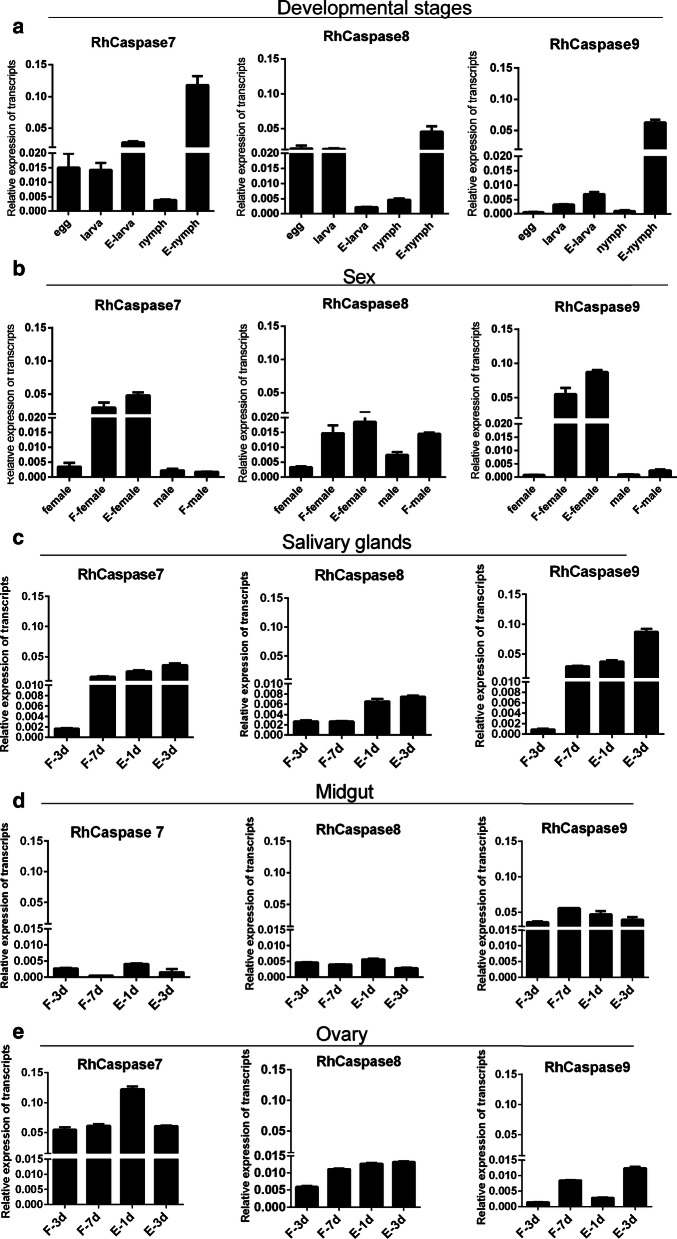
Transcription analysis of RhCaspases 7, 8 and 9 in *Rhipicephalus haemaphysaloides.***a** RT-qPCR analysis of RhCaspase 7, 8 and 9 gene expressions during different development periods. **b** RT-qPCR analysis of RhCaspase 7, 8 and 9 gene expressions according to sex. **c–e** Transcription of RhCaspase 7, 8 and 9 genes in different tissues of *R. haemaphysaloides*. *Abbreviations*: F, fed; E, engorged

The cDNA of eggs, larvae (unfed and engorged), and nymphs (unfed and engorged) were subjected to RT-qPCR to evaluate the expression profiles of RhCaspase 7, 8 and 9 genes during the developmental stages (Fig. 5a). The cDNA of unfed adults (female and male), fed adults (female and male), and engorged female ticks were used to determine the sex-specific profiles of RhCaspase 7, 8 and 9 genes. The transcription level of RhCaspases 7, 8 and 9 increased after feeding (Fig. 5a). The expression of RhCaspase 7, 8 and 9 genes did not change in male ticks at different feeding times, but in female ticks the expression level of RhCaspase 7, 8 and 9 genes increased during feeding (Fig. 5b).

After microdissection, the cDNA of salivary glands, ovary and midgut of adult female ticks at different feeding times were analyzed for RhCaspase 7, 8 and 9 gene expression profiles. RhCaspase 7, 8 and 9 genes are expressed in different tissues during tick feeding (including the early feeding period, the fast feeding period, and the end of the feeding period). However, the different genes had different expression levels in different organs. The expression levels of the RhCaspase 7 gene in the salivary gland and ovary were higher than in the midgut. Similar to levels in the salivary gland of fully engorged ticks after feeding, the RhCaspase 9 gene had consistent high expression in the midgut during all feeding time periods. However, the RhCaspase 8 gene had a low expression level in all three tissues (Fig. 5c–e). The expression of the three RhCaspases increased in the salivary gland during feeding, which supports the role of apoptosis in the process of salivary gland degeneration (Fig. 5c).

**Fig. 6:**
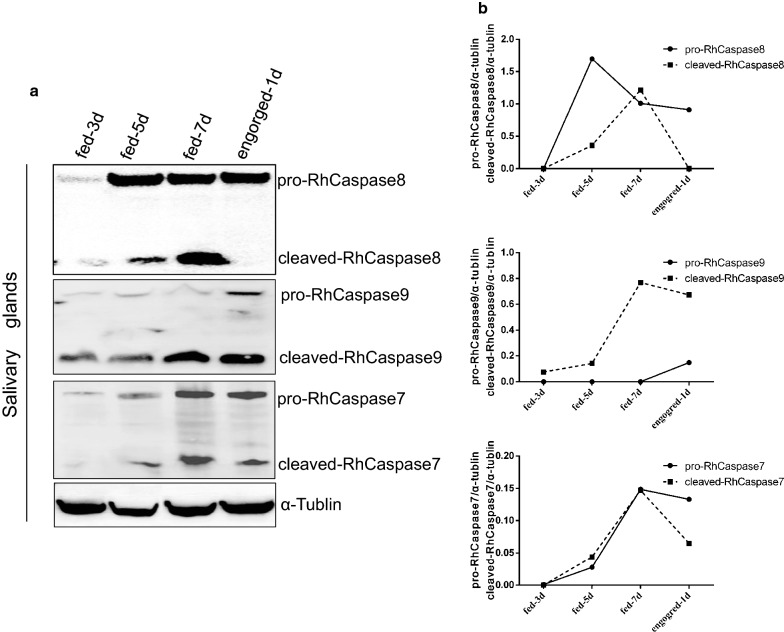
Western blot detection of RhCaspases 7, 8 and 9 of *Rhipicephalus haemaphysaloides* salivary glands at different feeding time points. **a** Tick salivary glands were screened with anti-RhCaspases 7, 8, and 9 sera, and anti-tubulin serum was used as a control. **b** Densitometry analysis of the western blot

Western blot analysis using anti-caspases sera showed that, in the salivary gland, caspases were cleaved during the feeding process. This is associated with salivary gland degeneration. Cleaved-RhCaspases 7 and 8 were detected in the fast feeding time (fed for 5 days to fed for 7 days) to post-engorgement and cleaved-RhCaspase 9 was detected after day 3 (Fig. 6). Caspase fragments matched the size of putative caspase fragments calculated by *in silico* analysis.

### RNAi of RhCaspases 7, 8 and 9

**Fig. 7:**
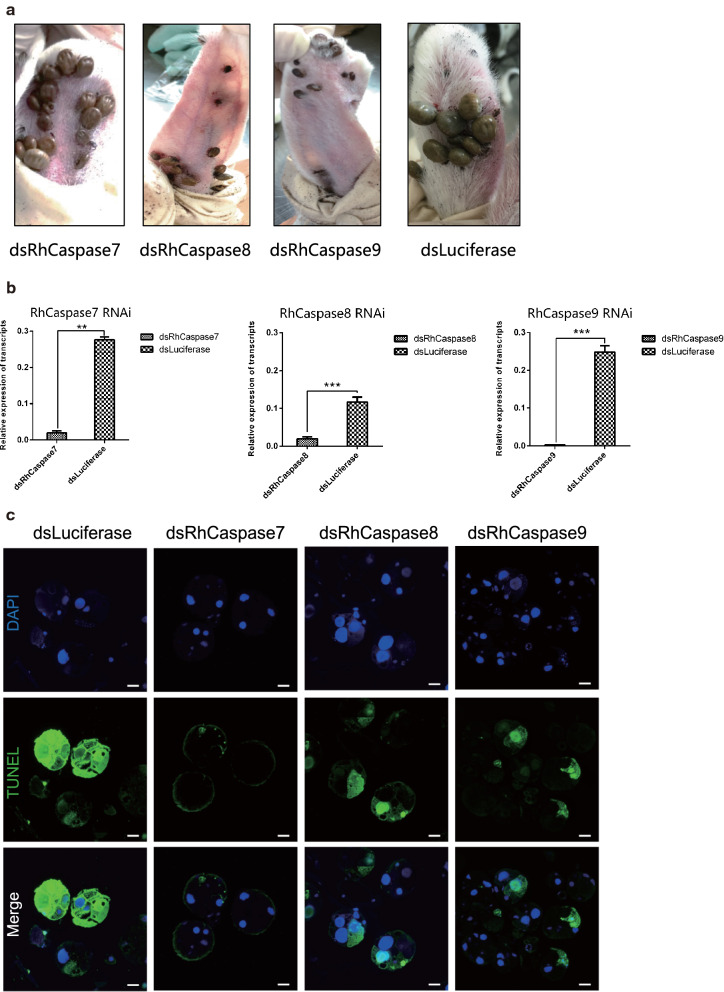
Biological effects of RNAi of *Rhipicephalus haemaphysaloides* RhCaspases 7, 8 and 9 *in vivo*. **a** Images comparing ticks inoculated with dsRhCaspases 7, 8 and 9 with control groups on day 6 of feeding. dsRhCaspase 9 inhibited the blood-feeding process and resulted in ticks smaller than the controls. **b** Confirmation of RhCaspase 7, 8 and 9 silencing using RT-qPCR. Total RNA was extracted on feeding day 5 from female ticks injected with dsRNA. Bars represent mean relative expression of RhCaspase 7, 8 and 9 genes; error bars represent standard error. ***P < 0.001, based on two-tailed Student’s t-tests. **c** Positive TUNEL staining decreased after RNAi of RhCaspases 7, 8 and 9, and DNA was stained with DAPI. *Scale-bars*: **c**, 50 μm

To identify function of RhCaspases 7, 8 and 9, the genes of RhCaspases 7, 8 and 9 were targets of RNA interference, *in vivo*. qRT-PCR analysis of ticks showed decreased levels of caspase mRNAs in caspase-dsRNA injected groups compared to luciferase-dsRNA injected controls (Fig. 7b) (RhCaspase 7 RNAi: *t*_(3)_ = 11.08, *P* = 0.0004; RhCaspase 8 RNAi: *t*_(3)_ = 33.64, *P* < 0.0001; RhCaspase 9 RNAi: *t*_(3)_ =15.16, *P =* 0.0001). Compared to the control groups, ticks injected with RhCaspase 9-dsRNA and RhCaspase 8-dsRNA showed a reduction in size on the sixth day after treatment and the effect was most obvious in the RhCaspase 9-dsRNA group (Fig. 7a). Consequently, the engorged weight of the caspase 9-dsRNA injected group was significantly lower than the control (Table 1). However, there was no significant difference in the attachment rate between the different experiment groups and control groups.

**Table 1:** Effect of knocking-down RhCaspases7, 8 and 9 on tick feeding behavior

Test group	dsRhCaspase 7	dsRhCaspase 8	dsRhCaspase 9	dsLuciferase
Attachment rate at 48 h (%)	88.22 ± 0.906	85.91 ± 1.256	87.56 ± 0.957	87.70 ± 0.690
Engorgement rate (%)	77.12 ± 2.692	73.157 ± 3.334	65.236 ± 4.369	75.79 ± 3.255
Engorged tick weight (mg)	377.1 ± 89.63	330.157 ± 40.158	220.157 ± 50.698	382.1 ± 57.92

TUNEL assays showed that the salivary glands of caspase-dsRNA injected groups (RhCaspases 7, 8 and 9) had reduced apoptosis levels compared with the control group (Fig. 7c).

### RhCaspase 7 can be cleaved by RhCaspases 8 and 9 *in vitro*

**Fig. 8:**
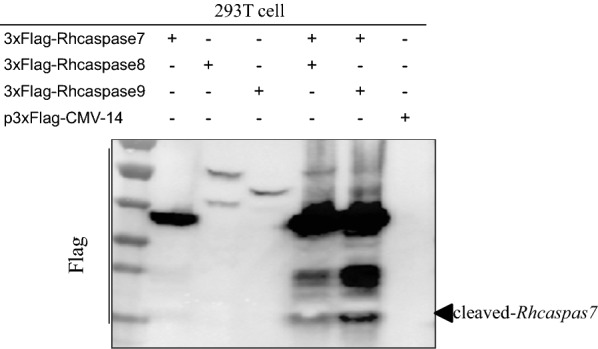
Western blot analysis of the interaction between RhCaspases 7, 8 and 9 by co-transfection into HEK-293T cells. Lane 1–3: cells transfected individually with p3×Flag-CMV-14-RhCaspase 7, 8 and 9 genes; Lane 4: cells co-transfected with p3×Flag-CMV-14-RhCaspase 7 and p3×Flag-CMV-14-RhCaspase 8; Lane 5: cells co-transfected with p3×Flag-CMV-14-RhCaspase 7 and p3×Flag-CMV-14-RhCaspase 9; Lane 6: cells co-transfected with p3×Flag-CMV-14

Co-transfection of HEK 293 cells with plasmids containing two different RhCaspase genes produced co-expression of the proteins. The results also showed that RhCaspase-7 is cleaved by the other two RhCaspases (Fig. 8). This confirms the *in silico* prediction that RhCaspase 7 is an executioner caspase and RhCaspases 8 and 9 are initiator caspases.

## Discussion

Tick salivary glands undergo degeneration after engorgement [4, 53, 54], and apoptosis has a role in this process [13, 14, 33]. *Rhipicephalus haemaphysaloides*, 1897 (Ixodida: Ixodidae) is a three-host hard tick widely distributed in China [55] and has been reported to be a vector of several pathogens [55–57]. We used an RNA-Seq approach to analyze gene expression differences in unfed and engorged *R. haemaphysaloides* salivary glands. The transcription level of apoptosis-related genes increased as a consequence of feeding. In addition, we found that apoptosis is involved in salivary gland degeneration.

Caspase genes were differently expressed in the salivary glands of unfed and engorged females. This was consistent with morphological [58, 59] and functional [5, 60] effects that occur in tick salivary glands during feeding. Many genes are activated by the feeding process and about 40% are upregulated. The expression of 40 apoptosis-related genes was affected by the feeding process. The majority of these apoptosis-related genes were upregulated, suggesting a relationship between the feeding process, salivary gland degeneration, and apoptosis.

Compared to other protein families, differentiation of the caspases is complex, since different species have several isoforms as well as a different number of genes. Also, caspases lack a standardized nomenclature, resulting in differences in nomenclature among species. However, we were able to identify and characterize three *R. haemaphysaloides* caspases, including two initiators (RhCaspases 8 and 9) and one executioner caspase (RhCaspase 7). Sequence analysis showed that RhCaspases 7, 8 and 9 have a conserved sequence characteristic of caspases. However, the two putative initiators, RhCaspases 8 and 9, do not contain the predicted CARD domain.

Although ticks are evolutionarily distant from insects, the phylogenetic analysis of RhCaspases from a variety of insects and other arthropod species suggests a close relationship with caspase homologs in insect species (Fig. 3c). Sometimes, the variability in the caspase nomenclature and number of caspase isotypes among different species makes it difficult to establish comparative relationships. For example, the *Drosophila* caspases are a well-studied model but the amino acid sequence of caspase DRONC is typical of caspase-2, but functionally similar to caspase-9 [61, 62]. Tick initiator caspases (RhCaspases 8 and 9) are closer to insect initiator caspases and executioner (RhCaspase 7) is more similar to insect executioner caspases.

RNAi is the most effective method for identifying gene functions in ticks [1]. Therefore, RNAi was used to study the physiological roles of the three identified RhCaspases. RhCaspase 9 silencing produced the most significant phenotype alteration in ticks during blood-feeding compared to RhCaspase 7, RhCaspase 8, and the control groups. The RT-qPCR results showed that RhCaspase 9 maintains a high level of transcription in the midgut during all feeding times and it is the most activated caspase in the salivary gland, as determined by western-blot. These data suggest that the role of RhCaspase 9 is related to blood-feeding. However, the precise biological function of RhCaspase 9 during tick feeding remains unclear. The tick salivary gland rapidly degenerates and disappears within 4 days after engorgement [53, 63]. TUNEL staining has been used to evaluate the rate of DNA fragmentation in degenerated tick salivary glands [13, 14]. Compared with the control group, the positive rate of TUNEL staining was significantly reduced by interfering with the RhCaspase 7, 8 and 9 gene expression using RNAi. Nevertheless, the dsRNAs for RhCaspases 7, 8 and 9 did not completely reduce the degeneration of tick salivary glands. This was expected, since there is redundancy in apoptosis pathways and other types of programmed cell death. In *D. melanogaster*, inhibiting caspase genes or the *atg8a* gene (autophagy related gene), is not sufficient to prevent salivary gland degradation. Only the simultaneous inhibition of both apoptotic and autophagic pathways impedes salivary gland histolysis [64].

Co-transfection of the cells with plasmids containing caspase genes was used to study the interaction between the different RhCaspases. Although co-transfection is a standard method used to analyze protein-protein interaction events *in vitro* [65], there are relatively few reports of this type of study in ticks. RhCaspase 7 is an executioner caspase, and RhCaspase 8 and 9 are initiator caspases [62, 66, 67].

## Conclusions

Three tick caspase molecules were identified in ticks and the roles of these caspases in tick physiology were studied. Our findings provide a basis for advanced studies on tick apoptosis. The knockdown of RhCaspase 9 *in vivo* inhibited blood-feeding of the tick, demonstrating that RhCaspase9 has the potential to become a candidate vaccine molecule.

## Supplementary information

**Additional file 1: Table S1.** Primers for *Rhipicephalus haemaphysaloides* caspase genes. **Table S2.** Primers used for quantitative real-time polymerase chain reactions (qPCRs) of *Rhipicephalus haemaphysaloides* caspase genes. **Table S3.** Primers for *Rhipicephalus haemaphysaloides* caspase ORF cloning *. **Table S4.** Primers for RNAi of *Rhipicephalus haemaphysaloides* caspase genes.

**Additional file 2: Figure S1.** GO terms enrichment analysis for unfed and engorged tick salivary glands. **Figure S2.** Pheatmap-plot for unfed and engorged tick salivary glands. **Figure S3.** Phylogenetic tree of *R. haemaphysaloides* caspases compared to the caspases of *Drosophila melanogaster*. Bootstrap values of 500 simulations are shown at the branches.

**Additional file 3: Alignment S1.** Sequences of unigenes (FASTA format).

**Additional file 4: Dataset S1.** Differentially expressed genes in salivary glands of unfed and engorged ticks.

## Data Availability

The datasets supporting the conclusions of this article are included within the article and its additional files.

## Abbreviations

TBPs: tick-borne pathogens
PCD: programmed cell death
PcAb: polyclonal antibodies
RT-qPCR: reverse transcription quantitative PCR
PBS: phosphate-buffered saline
TBS: Tris-buffered saline
GO: Gene: Gene Ontology
KOG: euKaryotic Orthologous Groups
KEGG: Kyoto Encyclopedia of Genes and Genomes
SDS-PAGE: sodium dodecyl sulfate polyacrylamide gel electrophoresis
IPTG: isopropyl β-D-1-thiogalactopyranoside
DAPI: 4’, 6’-diamidino-2-phenylindole
TUNEL: terminal deoxynucleotidyl transferase dUTP nick-end labeling
