# Supplementary material for: Initiator and executioner caspases in salivary gland apoptosis of Rhipicephalus haemaphysaloides
Source: Parasit Vectors. 2020 Jun 5;13:288. doi: 10.1186/s13071-020-04164-5 (PMC7275347; doi:10.1186/s13071-020-04164-5)
Supplement: Supplementary file 1 — Additional file 1: Table S1. Primers for Rhipicephalus haemaphysaloides caspase genes. Table S2. Primers used for quantitative real-time polymerase chain reactions (qPCRs) of Rhipicephalus haemaphysaloides caspase genes. Table S3. Primers for Rhipicephalus haemaphysaloides caspase ORF cloning *. Table S4. Primers for RNAi of Rhipicephalus haemaphysaloides caspase genes. [file 13071_2020_4164_MOESM1_ESM.docx]

**Additional file 1: Table S1** Primers for *Rhipicephalus haemaphysaloides* caspase genes

| Primer name | Primer sequence |
| --- | --- |
| RhCaspase-7-S | GTTTTCGTGGCAGGCTGTTCAAG |
| RhCaspase-7-A | GGATGGCTATGTGGCTAAGAGTAAG |
| RhCaspase-8-S | GGGGTTCTTGTTAAAGTTGCC |
| RhCaspase-8-A | TCCACTGAGGATATATGGATAAGAG |
| RhCaspase-9-S | TACCTATGTACACAGCAGCACTCC |
| RhCaspase-9-A | TGGCTGGTAGTCAGTAAAGAATCCC |

**^a^**S, forward primer; A, reverse primer

**Additional file 1: Table S2** Primers used for quantitative real-time polymerase chain reactions (qPCRs) of *Rhipicephalus haemaphysaloides* caspase genes.

| Primer name | Primer sequence |
| --- | --- |
| ELF1A-S | CGTCTACAAGATTGGTGGCATT |
| ELF1A-A | CTCAGTGGTCAGGT TGGCAG |
| RhCaspase-7-S | CTCAGCGAGCGAAGGGGCACGGACA |
| RhCaspase-7-A | AGCAGACTGGGACAGACATCTCCGT |
| RhCaspase8-S | CGCCACAGTTTGGGACCACAGGA |
| RhCaspase8-A | CTTCGCCTTTCACCTGTGCCCCT |
| RhCaspase9-S | GCTGACAAGCCCACTGGCGAACAAC |
| RhCaspase9-A | CATTCAGAGCAGAGTCAGCAGTCCG |

**^a^**S, forward primer; A, reverse primer**Additional file 1: Table S3** Primers for *Rhipicephalus haemaphysaloides* caspase ORF cloning *

| Primer name | Primer sequence |
| --- | --- |
| *RhCaspase7*-pET-28a-S | GGCTGATATC*GGATCC*ATGGCCGGCATGAGTGGGGA |
| *RhCaspase7*-pET-28a-A | GTGCGGCCGC*AAGCTT*GAGAGTAAGCCGCTCCTGGTTTCG |
| *RhCaspase8*-pET-28a-S | CTTCCTTCCCAGGTACAGGGCAATGGCGGTCGCTTGCAGTCCT |
| *RhCaspase8*-pET-28a-A | GTGCGGCCGC*AAGCTT*CTTCCTTCCCAGGTACAGGGCA |
| *RhCaspase9*-pET-28a-S | GGCTGATATC*GGATCC*ATGCCTGCAGGTCGACGATTTACC |
| *RhCaspase9*-pET-28a-A | GTGCGGCCGC*AAGCTT*G*GAGATTTGGCTGGTAGTCAGTAAAGAATC* |
| *RhCaspase7*-p3×Flag-CMV-14-S | TGAACCGTCAGAATTAAGCTTATGGCCGGCATGAGTGGGGA |
| *RhCaspase7*-p3×Flag-CMV-14-A | CTTTGTAGTCAGCCCGGGATCCAGAGTAAGCCGCTCCTGGTTTCG |
| *RhCaspase8*-p3×Flag-CMV-14-S | TGAACCGTCAGAATTAAGCTTATGGCGGTCGCTTGCAGTCCT |
| *RhCaspase8*-p3×Flag-CMV-14-A | CTTTGTAGTCAGCCCGGGATCCCTTCCTTCCCAGGTACAGGGCA |
| *RhCaspase9*-p3×Flag-CMV-14-S | TGAACCGTCAGAATTAAGCTTATGCCTGCAGGTCGACGATTTACC |
| *RhCaspase9*-p3×Flag-CMV-14-A | CTTTGTAGTCAGCCCGGGATCC*GAGATTTGGCTGGTAGTCAGTAAAGAATC* |

**^a^**S, forward primer; A, reverse primer

**Additional file 1: Table S4** Primers for RNAi of *Rhipicephalus haemaphysaloides* caspase genes

| Primer name | Primer sequence |
| --- | --- |
| *RhCaspase7* dsRNA-S1 | GGATCCTAATACGACTCACTATAGGGGAGCATTTTTGGGGGAATCTTTGG |
| *RhCaspase7* dsRNA-A1 | GGTCTTGGAGCACCGAGCAGAGGGC |
| *RhCaspase7* dsRNA-S2 | GGAGCATTTTTGGGGGAATCTTTGG |
| *RhCaspase7* dsRNA-A2 | **GGATCCTAATACGACTCACTATAGG**GGTCTTGGAGCACCGAGCAGAGGGC |
| *RhCaspase8* dsRNA-S1 | **GGATCCTAATACGACTCACTATAGG**AGTTTGGAGCCTCGGTTGAC |
| *RhCaspase8* dsRNA-A1 | GGGATACCGTGGCACTGTTT |
| *RhCaspase8* dsRNA-S2 | AGTTTGGAGCCTCGGTTGAC |
| *RhCaspase8* dsRNA-A2 | **GGATCCTAATACGACTCACTATAGG**GGGATACCGTGGCACTGTTT |
| *RhCaspase9* dsRNA-S1 | **GGATCCTAATACGACTCACTATAGG**CGGCTCCCCCATTTAGTAGATT |
| *RhCaspase9* dsRNA-A1 | CTTCTTCCGCCAGCCTCGC |
| *RhCaspase9* dsRNA-S2 | CGGCTCCCCCATTTAGTAGATT |
| *RhCaspase9* dsRNA-A2 | **GGATCCTAATACGACTCACTATAGG**CTTCTTCCGCCAGCCTCGC |

**^a^**S, forward primer; A, reverse primer, the sequence in bold underlined indicates the sequence of the T7 promoter .
