# Supplementary material for: Initiator and executioner caspases in salivary gland apoptosis of Rhipicephalus haemaphysaloides
Source: Parasit Vectors. 2020 Jun 5;13:288. doi: 10.1186/s13071-020-04164-5 (PMC7275347; doi:10.1186/s13071-020-04164-5)

**Additional file 2: Figure S1.** GO terms enrichment analysis for unfed and engorged tick salivary glands.


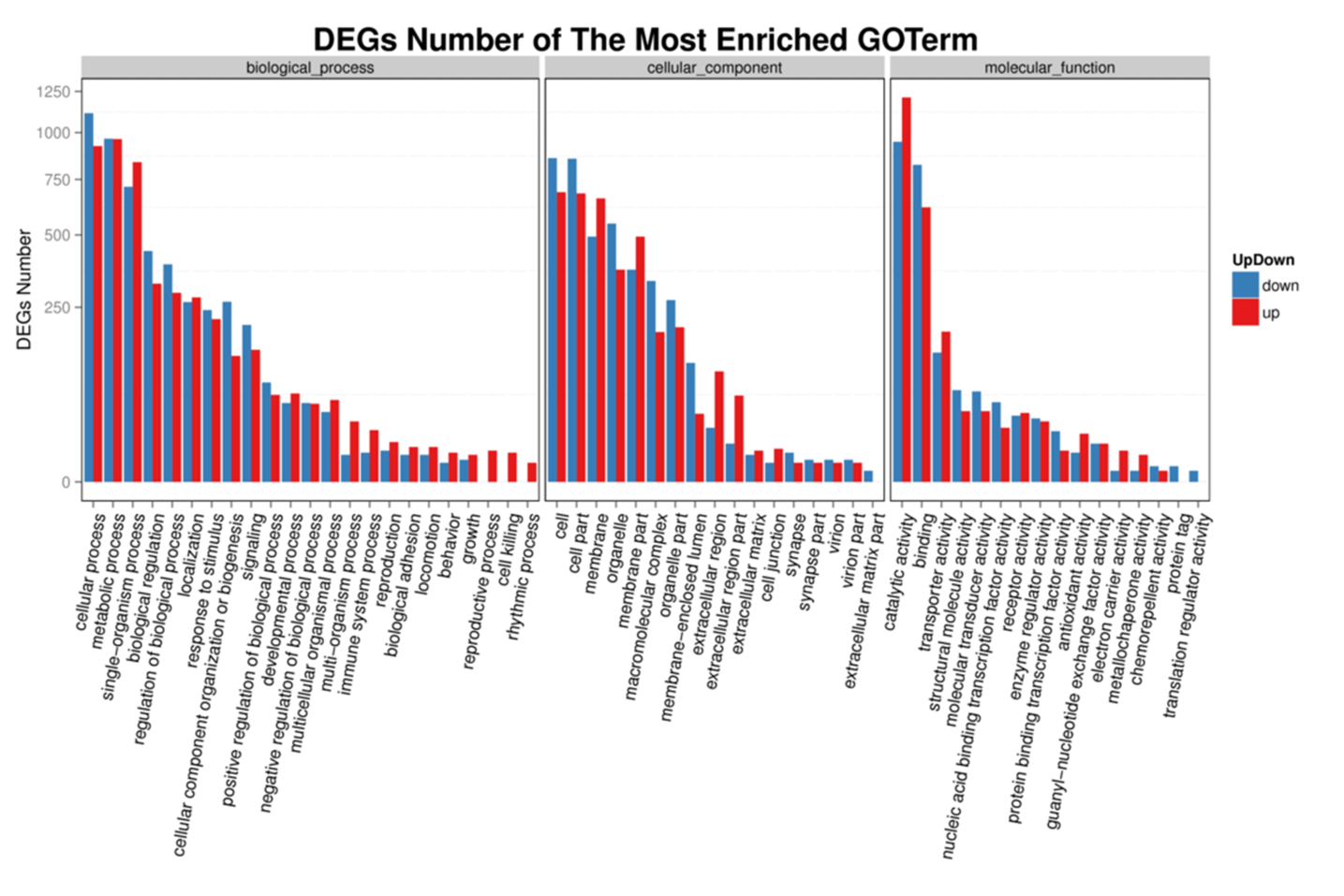


**Additional file 2:
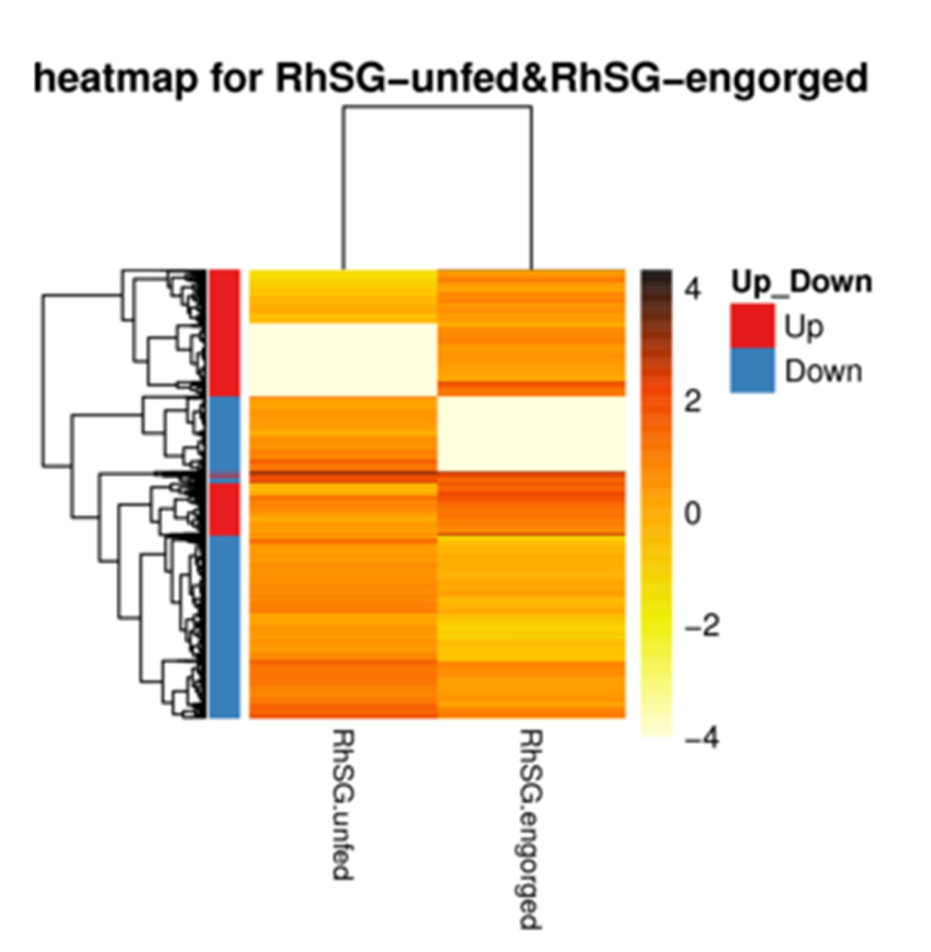
Figure S2.** Pheatmap-plot for unfed and engorged tick salivary glands.

**Additional file 2: Figure S3.** Phylogenetic tree of *R. haemaphysaloides* caspases compared to the caspases of other *Drosophila melanogaster*. Bootstrap values of 500 simulations are shown at the branches.


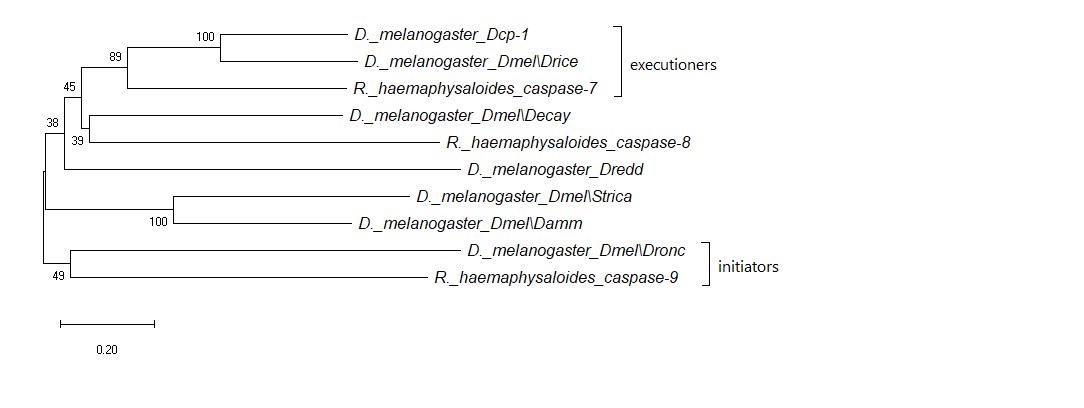

Supplement: Supplementary file 2 — Additional file 2: Figure S1. GO terms enrichment analysis for unfed and engorged tick salivary glands. Figure S2. Pheatmap-plot for unfed and engorged tick salivary glands. Figure S3. Phylogenetic tree of R. haemaphysaloides caspases compared to the caspases of Drosophila melanogaster. Bootstrap values of 500 simulations are shown at the branches. [file 13071_2020_4164_MOESM2_ESM.docx]
